# Self-Efficacy in Habit Building: How General and Habit-Specific Self-Efficacy Influence Behavioral Automatization and Motivational Interference

**DOI:** 10.3389/fpsyg.2021.643753

**Published:** 2021-05-07

**Authors:** Marco Stojanovic, Stefan Fries, Axel Grund

**Affiliations:** ^1^Department of Psychology, Bielefeld University, Bielefeld, Germany; ^2^Dr. phil. habil. Axel Grund Research Scientist Luxembourg Centre for Educational Testing – LUCET Faculty of Humanities, Education and Social Sciences, University of Luxembourg, Luxembourg, Luxembourg

**Keywords:** habit formation, specific self-efficacy, general self-efficacy, motivational interference, self-regulation, automaticity, app intervention

## Abstract

In this article, we investigate the role of self-efficacy (SE) in intentional habit building. We analyzed event sampling data from a habit building app we created that helps define and track habit data. We used hierarchical growth curve modeling and multilevel mediation to test our hypotheses. In a first study, *N* = 91 university students built new study habits over a period of 6 weeks in a controlled study. We found that the trait-like (Level 2) general self-efficacy predicted automaticity (i.e., habit strength) but not the experience of motivational interference (MI). In a second study with real user data, *N* = 265 idiographic habits have been analyzed. The specific SE associated with these habits – habit-specific self-efficacy (Level 1, HSE) – was measured during habit formation. We found that lagged HSE predicted automaticity and that lagged automaticity predicted HSE, indicating a positive feedback mechanism in habit building. Furthermore, we found that lagged HSE predicted less MI during habit performance. A multilevel mediation analysis showed significant effects of lagged HSE (Level 1) and aggregated HSE (Level 2) on MI, which were both partially mediated by automaticity. These results show the importance of defining the specificity of SE beliefs and how they interact with automaticity in the habit building process.

## Introduction

…we must make automatic and habitual, as early as possible, as many useful actions as we can… The more details of our daily life we can hand over to the effortless custody of automatism, the higher mental powers of mind will be set free for their own proper work.

– William James

Whether you think you can or you cannot, you are right.

– Henry Ford

Self-regulated learning can be very demanding. Students often report experiencing motivational interference (MI) during self-regulated learning (Grund et al., 2015), which is a mixture of bad mood, distractibility, thinking about alternative activities, task switching and low persistence, due to foregone alternative activities (Hofer and Fries, 2016). William James and Henry Ford point toward two factors that can alleviate the common emergence of MI during self-regulated behavior: Habits and self-efficacy (SE).

Acting habitually means acting automatically. More specifically, habits are automized behavioral patterns that get instigated through context-dependent triggers (Ouellette and Wood, 1998; Lally et al., 2010; Gardner et al., 2011; Verplanken, 2018). The more often a habit is executed, the more automatic it becomes (Lally et al., 2010; Stojanovic et al., 2020). When long-term behavioral change is to be induced, a promising approach is to establish good habits. Habits are particularity strong in predicting behavior that has been shown frequently in the past (i.e., potentially automized behavior), while intention is a better predictor for less automized, infrequent behavior (Ouellette and Wood, 1998).

Self-efficacy is the belief in one’s capability to produce desired results through one’s behavior (Bandura, 1994). SE can be viewed as a general trait-like belief about one’s ability to successfully overcome problems by action [general self-efficacy (GSE)] and as a task-specific belief about one’s ability to successfully deal with a given task like studying for a test (Chen et al., 2001; Luszczynska et al., 2005b). In this article, we examine both GSE and habit-specific self-efficacy beliefs (HSE) and their effects on automaticity and MI in the context of habit building. We refer to HSE as a task- or activity-specific belief, that is closely tied to concrete behavioral patterns people intend to repeat regularly in order to achieve a valued long-term goal and not as a belief in one’s capability to form beneficial habits in general. Hence, a student might have a high HSE for her morning-running-habit, but a low HSE for her after-lunch-study-habit. As we point out below, SE beliefs are strong predictors for successful self-regulation in various domains.

The aim of this study is to analyze habit building (i.e., increasing behavioral automaticity) as a viable way to systematically decrease MI during self-regulated behavior while exploring the role of SE in this process. In order to accomplish this, we analyzed two datasets that contain longitudinal event sampling data about experiences during habit execution in a real-life setting. In both studies, people built new habits using an app that was specifically created for this research. In the first study, university students built study habits in the context of a controlled study. We already published results from the first dataset showing that automaticity increased with habit repetitions and that automaticity reduces MI (Stojanovic et al., 2020). In the present article, we will test GSE as a predictor for automaticity and MI. In the second study, we analyze real-life user data from a published version of the app in Apple’s AppStore (Stojanovic, 2019), which contain a broader variety of habits. In the second study, HSE was measured in each habit repetition. Combining these ecologically valid datasets, malleable variables (automaticity and specific SE), and an efficient app-based intervention method, this article aims at providing a solid groundwork for feasible, and effective reduction of the experience of MI in self-regulated behavior.

### Habits and Self-Efficacy: Facilitators in Self-Regulation

The two constructs habit and SE are fundamentally different in their conceptions. On the one hand, we have a cognitive belief that is altered by *thinking*. SE beliefs are forged by processing information from own performance experiences, vicarious experiences of others, verbal persuasion, and emotional responses (Bandura, 1994). On the other hand, habits are created by *doing*. Habits develop by repeating a similar behavioral pattern in the same context.

#### Habits

A habit is an automized behavioral pattern learned through context-dependent repetition and, once established, triggered by contextual cues (Ouellette and Wood, 1998; Lally et al., 2010; Gardner et al., 2011; Verplanken, 2018). Automaticity is the central criterion for developing habits. Automaticity can be seen as the key defining feature, the “,active ingredient‘ of habit-behavior relationships” (Gardner et al., 2012, p. 1). Orbell and Verplanken (2010) describe habit as “cue-contingent automaticity” (p. 1). Traditionally, automaticity entails four characteristics: Unawareness, non-intentionality, efficiency, and uncontrollability (Bargh, 1994). These characteristics describe the maximally possible automaticity, a hypothetical extreme. Realistic, practical expectations concerning the automatization of complex habits in a real world setting contain a lesser version of these characteristics. We cannot expect total unconscious control of the habit, but we can expect a shrinking need of controlled thought when executing a well-practiced habit (Galla and Duckworth, 2015, Study 1; Wood et al., 2002). We cannot expect the absolute absence of intention, but we can expect decreasing levels of determined intentions guiding the habitual behavior (Ouellette and Wood, 1998; Gardner et al., 2020). We cannot expect absolute efficiency without using any attentional resources, but more efficiency with less attentional resources consumed (Wood et al., 2002; Evans and Stanovich, 2013). We cannot expect an inevitable habit execution once it is triggered, but we can expect a rising probability of habit execution once the trigger is present (e.g., Wood et al., 2005; Neal et al., 2011). Habits will have these characteristics to the degree they are automized.

There are two important cue-response connections in a habit that will get automized during the habit forming process: The instigation cue (*habitual instigation*) and the cue-response connections within the habit (*habitual execution*; Gardner et al., 2016). The instigation cue is a context cue that initializes the habit (e.g., arriving at the library triggers a learning habit). The habitual execution can be seen as a web of cue-response associations that make up the actual behavior of the habit itself, in which the end of the former behavioral response constitutes the cue for the following behavioral response (e.g., “searching a place to study” cues “think of needed books,” which cues “get needed books,” which cues “screen table of contents of all books,” etc.). The sum of all behavioral responses constitutes the habitual execution and is tied together even tighter with each iteration of the habit by strengthening its associations. Especially in complex habits like study habits that require constant adaption to new input (the new learning material), the habitual execution consists of more cue-response connections and varies more in that a certain cue might trigger several viable responses. After having read one passage, one could look up unknown words, reread for clarity, summarize the passage, and continue with the next passage, etc. On the other hand, a simple habit like brushing teeth before going to bed does not need such flexibility in behavioral responses. Elaborating on the distinction between habit instigation and execution, Phillips et al. (2019) capture the varying levels of flexibility in habitual behavioral responses by defining the terms “higher order habit” and “lower order habit.” Like the described complex study habit, higher order habits are automatically instigated, but can be executed in various forms. In contrast, the simple tooth brushing habit would be classified as a lower order habit, as it is rigidly executed in the same manner after instigation. Congruent with these considerations, more complex habits seem to take longer to build automaticity (Verplanken, 2006). It is not clear if complex habits generally have a lower maximally attainable level of automaticity than simple habits or just take longer to build it, but there is evidence that even moderate levels of automaticity of relatively complex study habits lead to a significant reduction of MI (Stojanovic et al., 2020).

This nature of habits – automized behavior – leads to needing less self-regulation during activities that would otherwise consume more self-regulatory resources. Gardner et al. (2011) found a medium-to-strong effect of habits on dietary behavior and physical activity in their meta-analysis. Concerning study related behavior, habit strength is associated with the ability to study under difficult circumstances, higher classroom engagement, homework completion, and GPA in college (Galla and Duckworth, 2015). Neal et al. (2013) showed that people even “fall back” into their good (and bad) habits when willpower is depleted – for example students during a phase in which they have several exams. In cases like this, strong learning habits can be a very useful default response. Once established, habits require less intention and less deliberate thought (Ouellette and Wood, 1998). Accordingly, changes in intentions have a far bigger impact on actually changing behavior when the behavior is *not* habitual (Webb and Sheeran, 2006).

A note on terminology. When investigating the formation of new habits over time, change is the object of scrutiny. While constructs like SE beliefs are existent from the beginning of the investigation, only varying in strength, a new “habit” that is to be formed, technically cannot exist, as behavioral repetition is necessary to establish a habit. Over time and with repetition, the behavior can obtain qualities that are used in modern scientific definitions to define the construct “habit”. In order to make communication about the development of habits in our research easier, we decided to call the precisely defined behavior participants set out to turn into habits in the scientific sense, “habits” from the beginning on. Hence, in the communication with the participants, we refer to the planned behavior as their “habit” and also when reporting results in the present article, we use this term, even though the behavior technically cannot be a habit in the scientific sense after the first repetition.

#### Self-Efficacy

Self-efficacy is also strongly tied to self-regulation. Here, the empirical basis is much broader than in habit research. There are strong correlations between GSE and self-regulation across different cultures (Luszczynska et al., 2004, 2005a). In the realm of academic achievement, SE predicts a higher GPA partially mediated by effort regulation (Komarraju and Nadler, 2013), and meta-analytic results show that academic SE is a strong predictor for GPA and a moderate one for college retention (Robbins et al., 2004). Performance SE belongs to one of the variables most strongly associated with achievement in higher education (Schneider and Preckel, 2017). Further meta-analyses found that SE has effects on health related intentions (strong effect), behavior (moderate effect), and is negatively related to a lack of feeling of accomplishment in the work context (Sheeran et al., 2016; Shoji et al., 2016).

On one hand, SE beliefs can vary significantly within individuals across domains, on the other hand, there is evidence that domain-specific SE beliefs are related to a higher order factor – GSE. Weak to moderate but consistent correlations between GSE and specific SE beliefs were found in various domains: In health-related behavior for exercise SE, nutritional SE, and smoking abstinence SE (Luszczynska et al., 2005b), or in cognitive tasks for exam SE and occupational tasks in different domains (e.g., art, literature, science, and mechanical; Chen et al., 2001). Hence, when investigating the influence of a belief such as SE with only weak to moderate connections between the higher order factor and its domain-specific equivalents, it seems necessary to investigate not only GSE but also specific SE.

Note that automaticity and SE facilitate self-regulated behavior, but do not act as triggers for the behavior *per se*. Automaticity needs a context cue to unfold. SE beliefs need to be confronted with activities to promote behavior. A student might cease to perform a highly automized, high-SE study habit after having attained her college degree. In the context of intentional (beneficial) habit building, we investigate the formation of automaticity and HSE with participants having set themselves an overarching, long-term goal, which will be present during the process.

### The Virtuous Cycle of Automaticity and Self-Efficacy

In research on habit building, we are interested in the question on how habits can be build as quickly and as solidly as possible. Automaticity and SE are strong and malleable predictors for desired behavior, making them predestined for interventions, but differ fundamentally in their underlying mechanisms in affecting behavior: Unconscious automaticity vs. deliberate efficacy beliefs. Wäschle et al. (2014) found that SE and study goal achievement have a reciprocal relationship resulting in a self-amplifying feedback loop or virtuous cycle: SE grows with successful study goal achievement, but declines with missed study goals. At the same time, it is a predictor of study goal achievement. SE is antecedent and effect of successful self-regulated learning. As creating and executing a new beneficial habit is nothing else than following a planned, self-regulated behavior in a stable context over time, we expect to find the same pattern in habit building: High GSE will predict successful habit building and HSE will grow in a positive feedback loop with automaticity of behavior, which will grow with successful habit repetitions. According to Bandura (1994), mastery experiences constitute the most important informational source for forming SE beliefs. The more often a habit is repeated, the more the behavioral sequence is automized, the more it becomes an act of mastery and should thus contribute to building a positive HSE.

We made the point earlier that it has to be accounted for specificity when investigating SE. GSE tends to be relatively stable over time with retest reliabilities of *r* = 0.60 over a 2-month period (Chen et al., 2001), *r* = 0.64–0.65 over a 2-year period (Schwarzer and Jerusalem, 1999), and *r* = 0.29 over 9 years (Grevenstein and Bluemke, 2015). Being more malleable in nature, specific SE is, unsurprisingly, more predictive for performance in the specific task (e.g., exam specific SE and exam performance, Chen et al., 2001). Accordingly, investigating a dynamic virtuous cycle, we paired the quickly increasing automaticity with the more change-sensitive HSE rather than the trait-like GSE.

### Hypotheses

We derived two sets of hypotheses from these considerations. In the first set, we make predictions about GSE. We hypothesize that a high GSE is beneficial for habit building (i.e., automatization; H1a) and reduces MI (H1b). We further expect the effect of GSE on MI to be mediated by automaticity (H1c).

**H1a**: GSE predicts automaticity.
**H1b**: GSE predicts MI.
**H1c**: Automaticity mediates the effect of GSE on MI.

In the second set, we make fundamentally the same predictions about HSE, albeit extended for the virtuous cycle assumption (H2a-b). Note that, as HSE was measured after each habit repetition as automaticity and MI, we could postulate hypotheses with time lagged predictors.

**H2a**: HSE*_*t*_*_–__1_ predicts automaticity*_*t*_*.
**H2b**: Automaticity*_*t*_*_–__1_ predicts HSE*_*t*_*.
**H2c**: HSE*_*t*_*_–__1_ predicts MI*_*t*_*.
**H2d**: Automaticity mediates the effect of HSE on MI.

## The Present Study

Two datasets were analyzed to test the postulated hypotheses. The first dataset stems from a longitudinal habit building study in which students used an app to build new study habits. Analyzes on this dataset have already been published (Stojanovic et al., 2020), but the role of the trait variable GSE (Level 2) for automaticity and MI has not yet been analyzed. The habit builder app that was used in this study was optimized and published on Apple’s AppStore (Stojanovic, 2019). The second study therefore contains real-life user data. In this second study, HSE was measured repeatedly during the habit building process (Level 1).

## Study 1: Habit Building and General Self-Efficacy

### Materials and Methods

#### Participants

In the study from Stojanovic et al. (2020), *N* = 91 university students (*M*_*age*_ = 22.3, *SD_*Age*_* = 4.9; 79.1% female) participated in return for course credit. Participants with at least 20 habit repetitions were registered for an additional lottery for a new iPad worth 500 Euros (∼600 United States dollars). Participants who did not log at least one habit repetition were excluded from the data analysis. The participants were recruited in psychology lectures of two German universities.

#### Procedure and Measures

Stojanovic et al. (2020) created a new iPhone app for the purpose of the study. It guided the participants through a pretest, the process of defining a new study habit, and was used to track habit data. GSE, automaticity, and MI were measured on a 11-point scale (from *0 = doesn’t apply at all* to *10 = applies perfectly*). GSE was assessed with 10 items in a pretest (Schwarzer and Jerusalem, 1999; e.g., “I can always manage to solve difficult problems if I try hard enough.”). After the pretest, the app led the participants through a habit definition process, in which they defined (1) *What* their habit would be (it had to be a new study habit for university), (2) *When* they planned to perform their new habit (e.g., after brushing teeth in the evening), (3) *how long* they planned to perform their habit (it had to be between 10 and 30 min), and (4) what their *goal* was for one habit repetition (e.g., summarize one lecture or read five pages).

Having finished this, the 6-week long event sampling phase started. Participants were instructed to answer the items the app presented them after they confirmed having completed their daily habit repetition. Automaticity was measured with six automaticity items from the Self-Report Habit Index (SRHI; Verplanken and Orbell, 2003; Lally et al., 2010; e.g., “My habit is something I do automatically.”). Experience of MI was measured with five items covering the five facets *mood* (“I was annoyed by my habit.”), *distractibility* (“My thoughts constantly digressed.”), *thoughts about alternatives* (“From time to time I thought about other things I let slide.”), *task switching* (“I switched between different activities.”), and *persistence* (“It was difficult to finish my habit.”). The persistence item was adapted from Brassler et al. (2015), while four remaining items were adapted from Grund et al. (2014). Automaticity, MI, and the degree of goal attainment (value between 0 and 100%) of their previously set habit repetition goal was measured after each habit repetition. Participants could see their automaticity scores plotted on a line graph in the app to see their habit development over time.

#### Data Analysis

The study contains hierarchical data with habit repetitions (Level 1) nested in persons (Level 2). We modeled growth curve models with multilevel regressions (Field, 2013) using IBM SPSS 25 to test H1a-b. We used maximum likelihood to estimate the parameters to be able to compare the different nested growth curve models for model fit.

All growth curve models (Model 1–9) describe the trajectories of the dependent variables automaticity, HSE or MI over time (i.e., habit repetitions). Models 1–4 are fitted to this first dataset and contain random intercepts (*u*_0_), random slopes (*u*_1_), the covariance of the random intercepts, and the random slopes [COV(*u*_0_,*u*_1_)] and Model 2 and 4 have a Level 2, person-level predictor (GSE). We specified unstructured random effects covariance matrices for Models 1–4.

Model 1 (Eq. 1) predicts automaticity at time *t* for person *p* with a random intercept *b*_0,_*_*p*_*, which is the average intercept of the sample *b*_00_ plus the individual deviation from that intercept *u*_0,_*_*p*_* (Eq. 2), plus the individual slope *b*_1,_*_*p*_*, which is the average slope for the effect of habit repetition (i.e., time) of the whole sample *b*_10_ plus the individual deviation from that slope *u*_1,_*_*p*_* (Eq. 3), times habit repetition plus habit repetition squared (habit repetition sq_*t,p*_) with a fixed beta (*b*_2_), which adds a quadratic trend, plus error_*t*,__*p*_. For Model 2–4, *b*_0,_*_*p*_* and *b*_1,_*_*p*_* are the same as in Model 1.

(1) $$\begin{array}{l} Automaticity_{t,p}=b_{0,p}+b_{1,p}Habit\;repetition_{t,p}\\ \;+b_{2}Habit\;repetition\;sq_{t,p}+{\text{ε}}_{t,p}. \end{array}$$

(2) $$b_{0,p}=b_{00}+u_{0,p}.$$

(3) $$b_{1,p}=b_{10}+u_{1,p}.$$

In Model 2 (H1a), we added the Level 2 predictor GSE, resulting in Eq. 4.

(4) $$\begin{array}{l} Automaticity_{t,p}=b_{0,p}+b_{1,p}Habit\;repetition_{t,p}\\ \;+b_{2}Habit\;repetition\;sq_{t,p}+b_{01}GSE+{\text{ε}}_{t,p}. \end{array}$$

Model 3 predicts MI at time *t* for person *p* with habit repetition (i.e., time) plus error*_*t*,__*p*_*.

(5) $$M\,I_{t,p}=b_{0,p}+b_{1,p}\,H\,a\,b\,i\,t\,r\,e\,p\,e\,t\,i\,t\,i\,o\,n_{t,p}+\varepsilon_{t,p}.$$

In Model 4 (H1b), we added GSE at Level 2, resulting in Eq. 6.

(6) $$M\,I_{t,p}=b_{0,p}+b_{1,p}\,H\,a\,b\,i\,t\,r\,e\,p\,e\,t\,i\,t\,i\,o\,n_{t,p}+b_{01}\,G\,S\,E+\varepsilon_{t,p}.$$

### Results

#### Preliminary Findings

The dataset from Stojanovic et al. (2020) contained *N* = 2,574 habit repetitions of *N* = 91 participants. On average, a participant completed *M* = 28.29 (*SD* = 17.88) habit repetitions. *n* = 57 participants (62.6%) logged at least 21 habit repetitions, *n* = 27 (29.7%) logged at least 42 habit repetitions and *n* = 21 (23.1%) kept logging data even after the 6-week period they were asked to enter data. The case with the most habit repetitions contained 69 measurement points. The last-mentioned 21 cases were included into the data analysis with all their respective data points^1^. Concerning pauses between habit repetitions (i.e., not doing a habit repetition for at least 1 day), with *n* = 1,953 (78.7%) the majority of habit repetitions were done without pause, *n* = 336 (13.5%) were done with a pause of 1 day, and *n* = 194 (7.9%) were done with a pause of 2 days or more. The participants indicated a high average degree of goal attainment (i.e., the attainment of the goal set for one habit repetition) of *M* = 83.4% (*SD* = 21.46) with a median of 91%. The participants achieved 100% (vs. 0%) of their defined habit goal in 39.9% (vs. 0.3%) of the cases, indicating productive study behavior during the habit repetitions.

#### Automaticity and General Self-Efficacy

H1a aims at testing GSE as a Level 2 predictor for automaticity in the habit formation process. In this section, we first describe the hierarchical automaticity baseline model, which represents habit formation over time as reported by Stojanovic et al. (2020) and then, in a new analysis for this article, add GSE to test H1a.

##### Automatization over time

To model habit formation over time, automaticity was predicted with habit repetition as the time variable with random slopes and random intercepts. Furthermore, habit repetition was squared and added as a predictor to model decreasing automaticity gains in higher habit repetition ranges (see Table 1, Model 1). This pattern replicates a typical habit growth trajectory with steep automaticity gains at the beginning of the habit building process and asymptotically decreasing automaticity growth in the higher repetition range (Lally et al., 2010) and constitutes the automaticity baseline model (Model 1). Neither pauses between habit repetitions, nor age or gender had an influence on automaticity (Stojanovic et al., 2020).

**TABLE 1:** Multilevel regressions of automaticity and motivational interference on general self-efficacy based on controlled study data (Stojanovic et al., 2020).

Parameter	Model 1			Model 2 (H1a)			Model 3			Model 4 (H1b)		
	Automaticity						Motivational interference					
	Estimate	*SE*	95% CI	Estimate	*SE*	95% CI	Estimate	*SE*	95% CI	Estimate	*SE*	95% CI
**Fixed effects**												
Intercept (*b*_00_)	2.000***	0.194	1.615, 2.385	−1.485	0.847	−3.166, 0.196	3.771***	0.179	3.416, 4.126	4.416***	0.727	2.974, 5.858
**Level 1**												
Habit repetition (*b*_10_)	0.113***	0.009	0.095, 0.131	0.114***	0.009	0.096, 0.132	−0.049***	0.006	−0.063, −0.036	−0.050***	0.007	−0.063, −0.037
Habit repetition sq (*b*_2_)	−0.001***	<0.001	−0.0013, −0.0008	−0.001***	<0.001	−0.0013, −0.0008						
**Level 2**												
GSE (*b*_01_)				0.609***	0.145	0.322, 0.896				−0.113	0.124	−0.358, 0.132
**Random effects**												
Random intercept (VAR *u*_0_)	3.216***	0.498	2.374, 4.358	2.715***	0.422	2.002, 3.681	2.559***	0.428	1.843, 3.553	2.522***	0.423	1.815, 3.503
Cov. rand. intercept, rand. slope (COV *u*_0_, *u*_1_)	−0.031*	0.015	−0.0612, −0.0013	−0.030*	0.014	−0.0573, −0.0029	−0.051***	0.013	−0.0772, −0.0257	−0.051***	0.013	−0.0765, −0.0252
Random slope (VAR *u*_1_)	0.004***	<0.001	0.003, 0.007	0.004***	<0.001	0.003, 0.007	0.002***	<0.001	0.002, 0.004	0.002***	<0.001	0.002, 0.004

*Note.* All estimated coefficients are unstandardized. Variables as specified in the model equations are in parentheses. CI, confidence interval; Habit repetition sq, habit repetition squared; GSE, general self-efficacy; VAR, variance; COV, covariance; and Cov. rand. intercept, rand slope, covariation of the random intercept and the random slope. *p < 0.05 and ***p < 0.001.

##### Automatization and general self-efficacy

To test H1a, the influence of GSE on automaticity, GSE was added to the automaticity baseline model (Model 1) as a Level 2 predictor, resulting in Model 2. As expected, GSE predicted automaticity, *b* = 0.609 (*SE* = 0.145), *t*(97.36) = 4.21, and *p* < 0.001.

#### Motivational Interference and General Self-Efficacy

To test H1b, the influence of GSE on MI during habit repetition, we added GSE to a MI baseline model from Stojanovic et al. (2020) with random slopes, random intercepts, and habit repetition as its only predictor (Model 3), which resulted in Model 4 (see Table 1). Unexpectedly, GSE did not predict MI, *b* = −0.113 (*SE* = 0.126), *t*(99.64) = 0.91, and *p* = 0.363. With this finding, the mediation hypothesis H1c can be revoked as well as GSE fails to predict MI.

### Short Discussion

Study 1 generated two main findings. Firstly, GSE was associated with higher automaticity. Participants with higher GSE reported higher automaticity scores over a variety of individual study habits containing different learning activities such as reading, writing or rehearsal (for more details on the study habit contents, see Stojanovic et al., 2020). Secondly, contrary to our predictions, GSE was not associated with the experience of MI during habit performance. These results indicate that GSE might generally facilitate the intentional formation of habits. However, this broad, positive attitude toward one’s capability to deal with problems in general does not protect one against situation-specific motivational impairments. While the extensive applicability to different domains and kinds of behaviors is an advantage of the construct (see Luszczynska et al., 2005b), a drawback in terms of predictive power of the trait-like GSE belief, modeled here as a time-invariant covariate, is that it cannot capture dynamics of change that happen during the development of new habits. By increasing the specificity of SE, it changes conceptually from a general belief to a habit-specific attribute, from GSE to HSE, which is measurable as a time-variant variable we expect to develop and influence the habit building process significantly.

## Study 2: Habit Building and Habit Self-Efficacy

### Materials and Methods

#### Participants

The dataset we analyzed here stems from a refined version of the habit building app we used in our study earlier (Stojanovic et al., 2020), which was published in the Apple AppStore under the name “Grow – Habit Builder.” We analyzed user data from a total of *N* = 196 users, who tracked a total of *N* = 265 habits (Level 2) from 16.09.2018 to 14.08.2020, including *N* = 2,132 habit repetitions. Habits with less than two logged habit repetitions and data from the first author himself had previously been excluded from the dataset (529 cases). The majority of the data comes from Germany (29.1%), India (21.2%), and the United States (12.8%). The rest of the data points come from countries from all over the world with each contributing less than 4% of the total. 47.9% of users were female. 49.7% of the users were in the age group of 18–24 years, 32.0% were in the age group of 25–34 years, 13.0% were in the age group of 35–44 years, and 5.4% were in the age group of 45–54 years^2^.

#### Procedure and Measures

In the AppStore version of the habit builder app, the habit definition process was similar to that in Stojanovic et al. (2020), albeit with extensions (step 1, 2, 7, 8, and 9 were added) and minor adaptions for usability. In a first step, the users gave their habit a (1) *name*. Then, users were asked to define the (2) *long-term goal* they want to achieve with the defined habit. Concerning the (3) *how-long*-step, users were not constrained to a certain duration as in Stojanovic et al. (2020), but the app recommended a range of 3–60 min. Users were told that they should stop their habit only after having reached their habit action goal (step 6) and not after the defined duration. Defining a duration was supposed to help the users planning their habit and integrating it more easily in the flow of their day. Excluding 19 outlier habits with over 120 min as a duration goal, the average defined duration was *M*_duration_ = 23.61 (*SD*_duration_ = 23.71) with a median of *Mdn*_duration_ = 15. Then, users had to define a (4) *context* for their habit (formerly named “when” in Study 1) by specifying a time in the flow of the day (e.g., “after brushing teeth”) and a physical environment (e.g., “lying in bed”). Then, the core of the habit is the defined, the (5) *habit action* (formerly named “what” in Study 1). Users were asked to write down the specific action they would perform during their habit (e.g., “Write blog content” or “Learn new coding principle”). Note that users were not restricted to study habits as in Study 1. In the following step, users defined a (6) *habit action goal*, which would render a current habit repetition completed after achieving (e.g., “Write at least 200 words” or “Create a working code snippet with the new coding principle”). Next, users were asked to define a short version of their habit, which they should perform instead of the normal habit if they were under time pressure or feel they would not be able to perform their full habit for any other reason. The so-called (7) *emergency habit action* and the (8) *emergency habit action goal* were defined analogously to steps 4 and 5 but with the instruction to make it considerably shorter and be able to perform it anywhere if possible. 225 (10.6%) of the 2,132 analyzed habit repetitions were marked as emergency habit repetitions. As emergency habit repetitions were used sparingly and are similar to the normal version of the habit, emergency habit repetitions were analyzed as normal habit repetitions. In the (9) *frequency*-step, the users could choose the weekdays on which they wanted to perform the habit (i.e., were not restricted to daily habit repetitions). However, the app recommended setting the goal of performing the habit daily, which was done in 222 (83.8%) of the 265 analyzed habits. As a second part of this step, the users chose a time on which they wanted to be reminded of their new habit on the chosen weekdays. Finally, the app showed the users a summary of the habit and adaptions could be made for each step before saving the new habit.

Having finished this, the users could log data for their habit after each habit repetition. As we integrated more scales in the AppStore version of the app, we had to keep the questionnaire length short, because breakoff rates in mobile surveys rise with survey length (Mavletova and Couper, 2015). The event sampling process was the same as in Stojanovic et al. (2020), however, with the following adaption for efficiency and usability. Not all items for each scale were presented at each measurement point, but randomly selected items from each respective scale. That way, the users could quickly log their data with only 9 to 13 items in total, while maintaining the conceptual breadth of each scale over the long term. In mobile surveys, modularization of longer questionnaires in shorter chunks does not seem to reduce data quality and can even reduce item missings and satisficing (Toepoel and Lugtig, 2018). Among other variables not relevant for this article, automaticity, context stability, HSE, and MI were measured. The app was coded in a way that it was made sure that during the first habit repetitions, each item of each scale was answered at least once. Only after that the items would be selected randomly from the respective scales. Users answered the items using a 11-point scale (from *0 = strongly disagree* to *10 = strongly agree*). Context stability was measured with one item [“Compared to your earlier habit repetitions: How similar was the context of THIS habit repetition (environment, time of day, and people around you, etc.) to your usual habit context from earlier repetitions?”] after every habit repetition from the third one onward and answered on a *0–100%* scale.

##### Habit self-efficacy

Habit self-efficacy was measured from repetition three on with two randomly selected items, respectively. We used a scale of six items, which was adapted and extended from Schwarzer and Renner (2009, *The Physical Exercise Self-Efficacy Scale*, p. 7; e.g., “I can manage to stick to my habit, even when I have worries and problems.“).

##### Automaticity

Automaticity was measured with 10 partially adapted items from the SRHI^3^ (Verplanken and Orbell, 2003; e.g., “This habit is something I do automatically”). In the first five repetitions, automaticity was measured with 3–4 items and with two items in all following repetitions.

##### Motivational interference

Experience of MI was measured with five items as in Study 1 (Stojanovic et al., 2020). MI was measured on each repetition with 3–4 items.

Users could see their automaticity and MI value (recoded and called *motivational resilience* in the app) over habit repetitions on a chart in the app any time.

#### Data Analysis

In the user dataset, the habit repetitions (Level 1) are nested in habits (Level 2), which are technically again nested in persons (Level 3). However, as most users in the analyzed dataset only logged data for one habit (*n* = 150; 77%), Level 2 and Level 3 had such a big overlap that we could parsimoniously model the user data with Level 1 and Level 2. Furthermore, all relevant variance – coming from the person, the specific habit or the interaction of both – will automatically be represented by Level 2 parameters. So potential Level 3 variance will also be “caught” by Level 2 parameters. We modeled growth curve models with multilevel regressions (Field, 2013) using IBM SPSS 25 to test H1a-b and H2a-c. We used maximum likelihood to estimate the parameters to be able to compare the different nested growth curve models for model fit.

We tested our HSE-Automaticity-Mediation-Hypothesis H2d by conducting a multilevel mediation analysis with the MLmed Beta 2 SPSS macro by Rockwood (downloaded from https://njrockwood.com/mlmed; Rockwood, 2017). We specified random intercepts and used restricted maximum likelihood to estimate the parameters. In the multilevel mediation analysis, the mediation is tested on Level 1 and Level 2, meaning that each path will have a coefficient for each level of analysis. MLmed uses centered Level 1 variables to estimate within-effects. The between-effects of Level 2 are estimated by the mean values (e.g., the average MI value over all habit repetitions).

The user-data-models (Models 5–9) do not have any Level 2 predictors and only random intercepts as we found in preliminary analyses that random slopes were redundant in these models. Furthermore, *b*_0,_*_*p*_* is the same as in Model 1 for all Models. All coefficients in the growth curves as well as in the multilevel mediation are unstandardized.

Model 5 predicts automaticity at time *t* for person *p* with a random intercept *b*_0,_*_*p*_*, plus the effects of habit repetition (i.e., time; *b*_1_), habit repetition squared (*b*_2_), and habit pausing (*b*_3_) plus error*_*t*,__*p*_*. For Models 5–9, *b*_0,_*_*p*_* is the same as in Model 1 (Eq. 2).

(7) $$\begin{array}{l} \;Automaticity_{t,p}=b_{0,p}+b_{1}Habit\;repetition_{t,p}\\ +b_{2}Habit\;repetition\;sq_{t,p}+b_{3}Habit\;pausing_{t,p}+{\text{ε}}_{t,p}. \end{array}$$

In Model 6 (H2a), we added the time-lagged Level 1 predictor HSE*_*t*_*_–__1,_*_*p*_*, resulting in Eq. 8.

(8) $$\begin{array}{l} \;Automaticity_{t,p}=b_{0,p}+b_{1}Habit\;repetition_{t,p}\\ +b_{2}Habit\;repetition\;sq_{t,p}+b_{3}Habit\;pausing_{t,p}\\ \;+b_{4}HSE_{t-1,p}+{\text{ε}}_{t,p}. \end{array}$$

Model 7 predicts HSE at time *t* for person *p* with a random intercept *b*_0,_*_*p*_* plus habit repetition (i.e., time), habit repetition squared (*b*_2_), and habit pausing (*b*_3_), and time-lagged automaticity (automaticity*_*t*_*_–__1,_*_*p*_*) plus error*_*t*,__*p*_*.

(9) $$\begin{array}{l} \;HSE_{t,p}=b_{0,p}+b_{1}Habit\;repetition_{t,p}\\ +b_{2}Habit\;repetition\;sq_{t,p}+b_{3}Habit\;pausing_{t,p}\\ \;+b_{5}Automaticity_{t-1,p}+{\text{ε}}_{t,p}. \end{array}$$

Model 8 predicts MI at time *t* for person *p* with a random intercept *b*_0,_*_*p*_* plus habit repetition (i.e., time), automaticity, and error*_*t*,__*p*_*.

(10) $$\begin{array}{l} MI_{t,p}=b_{0,p}+b_{1}Habit\;repetition_{t,p}\\ \;+b_{2}Automaticity_{t,p}+{\text{ε}}_{t,p}. \end{array}$$

In Model 9 (H2b), we added HSE*_*t*_*_–__1,_*_*p*_*, resulting in Eq. 11.

(11) $$\begin{array}{l} \;MI_{t,p}=b_{0,p}+b_{1}Habit\;repetition_{t,p}\\ +b_{2}Automaticity_{t,p}+b_{3}HSE_{t-1,p}+{\text{ε}}_{t,p}. \end{array}$$

### Results

#### Preliminary Findings

On average, users logged *M* = 14.91 (*SD* = 18.84) habit repetitions for a habit. Dominant domains were study (e.g, reading and summarizing study material after the evening snack; learning Spanish with a language learning app before going to bed), exercise (e.g., doing pushups before going to work; doing video-guided yoga before breakfast) and mental focusing practices (e.g., meditating after lunch; doing breathing exercises while having a cold shower in the morning). Concerning pauses between habit repetitions (i.e., not doing a habit repetition for at least 1 day), with *n* = 1,190 (63.7%) the majority of habit repetitions were done without pause, *n* = 290 (15.5%) were done with a pause of 1 day, and *n* = 387 (20.7%) were done with a pause of 2 days or more. Users indicated a high average degree of context stability over *n* = 1,570 habit repetitions (context stability was not measured in the first two habit repetitions) of *M* = 81.40% (*SD* = 24.45) with a median of *Mdn* = 91%. Furthermore, users indicated a high average degree of goal attainment (i.e., the attainment of the goal set for one habit repetition) over *n* = 2.132 habit repetitions of *M* = 85.01% (*SD* = 25.88) with a median of *Mdn* = 100%. The participants achieved 100% (vs. 0%) of their defined habit goal in 62.5% (vs. 0.3%) of the cases, indicating productive habit behavior during the habit repetitions.

#### Automatization Over Time and Habit Pausing

We could replicate the automaticity baseline model with the user data (see Lally et al., 2010; Stojanovic et al., 2020) with habit repetition as a positive, linear predictor and habit repetition squared as a negative predictor, slowing automaticity gains down in high repetition ranges. In contrast to our earlier findings (Stojanovic et al., 2020), we found that habit pausing negatively predicted automaticity, which is why we added this predictor to the baseline model as a control variable, resulting in Model 5 (see Table 2).

**TABLE 2:** Virtuous-cycle-models of automaticity on habit-specific self-efficacy based on real-life app user data.

Parameter	Model 5			Model 6 (H2a)			Model 7 (H2b)		
	Automaticity						Habit self efficacy		
	Estimate	*SE*	95% CI	Estimate	*SE*	95% CI	Estimate	*SE*	95% CI
**Fixed effects**									
Intercept (*b*_00_)	3.762***	0.144	3.478, 4.045	1.898***	0.234	1.437, 2.359	3.800***	0.167	3.470, 4.130
**Level 1**									
Habit repetition (*b*_1_)	0.162***	0.009	0.145, 0.180	0.095***	0.010	0.075, 0.115	0.071***	0.009	0.053, 0.088
Habit repetition sq (*b*_2_)	−0.001***	<0.001	−0.0016, −0.0012	−0.001***	<0.001	−0.0011, −0.0006	−0.0005***	0.0001	−0.0007, −0.0003
Habit pausing (*b*_3_)	−0.008*	0.003	−0.0150, −0.0013	−0.011*	0.004	−0.019, −0.002	−0.010**	0.003	−0.016, −0.004
HSE*_*t*_*_–__1_ (*b*_4_)				0.416***	0.029	0.359, 0.472			
Automaticity*_*t*_*_–__1_ (*b*_5_)							0.327***	0.022	0.285, 0.370
**Random effects**									
Random intercept (VAR *u*_0_)	2.565***	0.334	1.979, 3.324	2.061***	0.389	1.424, 2.983	1.790***	0.308	1.277, 2.508

*Note.* All estimated coefficients are unstandardized. Variables as specified in the model equations are in parentheses. CI, confidence interval; HSE_*t–1*_, habit-specific self-efficacy value of the previous habit repetition; and VAR, variance. *p < 0.05, **p < 0.01, and ***p < 0.001.

#### Automatization and Habit Self-Efficacy: A Virtuous Cycle

**FIGURE 1:**
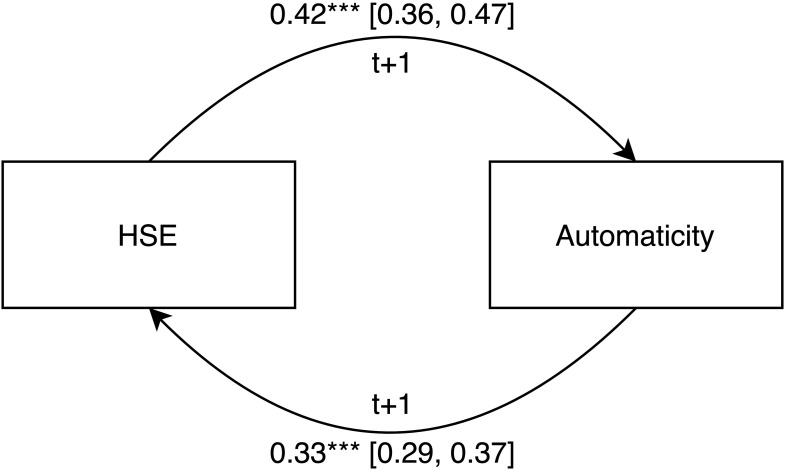
Virtuous cycle of automaticity and habit self-efficacy. *Note.* Virtuous cycle of habit self-efficacy (HSE) and automaticity with HSE at time t predicting automaticity at t + 1 and automaticity at t predicting HSE at t + 1. 95% confidence intervals are in brackets. ***p < 0.001.

H2a and H2b combined can deliver evidence for a virtuous cycle in which automaticity and HSE amplify each other in the habit building process leading to stronger habits. H2a aims at testing HSE as a Level 1 predictor for automaticity and in H2b we test if automaticity can in turn predict HSE. To test H2a, the time-lagged predictor HSE*_*t*_*_–__1_ was added to the controlled automaticity baseline model (Model 5) as a Level 1 predictor, resulting in Model 6 (see Table 2). So, the HSE-value from the previous habit repetition (*t*–1) was used to predict the current automaticity value at repetition t. As expected, HSE*_*t*_*_–__1_ predicted automaticity, *b* = 0.416 (*SE* = 0.029), *t*(1385.39) = 14.47, and *p* < 0.001. To test H2b, we defined a Model in which we predicted HSE with the time-lagged predictor automaticity*_*t*_*_–__1_ while keeping habit repetition, habit repetition squared, and habit pausing as predictors, resulting in Model 7 (see Table 2). So, the automaticity-value from the previous habit repetition (*t*–1) was used to predict the current HSE value at repetition *t*. As expected, automaticity*_*t*_*_–__1_ predicted HSE, *b* = 0.327 (*SE* = 0.022), *t*(1591.31) = 15.14, and *p* < 0.001. With the corroborating evidence for H2a and H2b, the data suggest a virtuous cycle of automaticity and HSE as depicted in Figure 1.

#### Habit Self-Efficacy Reduces Motivational Interference

To test H2c, the influence of HSE on MI during habit repetition, we first specified a simple baseline model with random intercepts and habit repetition and automaticity as predictors (Model 8). Then, we added HSE*_*t*_*_–__1_ to Model 8, resulting in Model 9 (see Table 3 for both models). As expected, HSE*_*t*_*_–__1_ predicted MI above habit repetitions and automaticity, *b* = −0.141 (*SE* = 0.025), *t*(1477.43) = −5.63, and *p <* 0.001.

**TABLE 3:** Multilevel regressions of motivational interference on automaticity and habit-specific self-efficacy based on real-life app user data.

Parameter	Model 8			Model 9 (H2c)		
	Motivational interference					
	Estimate	*SE*	95% CI	Estimate	*SE*	95% CI
**Fixed effects**						
Intercept (*b*_00_)	4.280***	0.119	4.046, 4.514	5.158***	0.185	4.795, 5.522
**Level 1**						
Habit repetition (*b*_1_)	−0.007*	0.003	−0.012, −0.001	–0.002	0.003	−0.008, 0.004
Automaticity (*b*_2_)	−0.269***	0.018	−0.304, −0.234	−0.288***	0.022	−0.331, −0.245
HSE*_*t*_*_–__1_ (*b*_3_)				−0.141***	0.025	−0.190, −0.092
**Random effects**						
Random intercept (VAR *u*_0_)	1.208***	0.185	0.895, 1.631	0.972***	0.201	0.648, 1.459

*Note.* All estimated coefficients are unstandardized. Variables as specified in the model equations are in parentheses. CI, confidence interval; HSE_*t–1*_, habit-specific self-efficacy value of the previous habit repetition; and VAR, variance. *p < 0.05 and ***p < 0.001.

#### Automaticity Mediates the Effect of Habit Self-Efficacy on Motivational Interference

**FIGURE 2:**
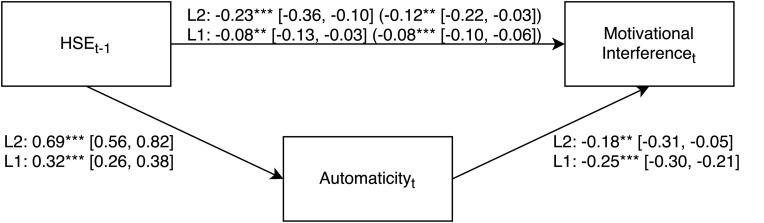
Multilevel mediation with automaticity mediating the effect of habit self-efficacy on motivational interference. *Note.* Multilevel mediation analysis with unstandardized regression coefficients of the effect of HSE_*t*__–__1_ (habit-specific self-efficacy from the previous habit repetition) on motivational interference through automaticity. The first coefficient on the path from HSE_*t*__–__1_ to motivational interference represents the direct effect without the mediator; the coefficient in parentheses on this path represents the indirect effect with the mediator included in the model. The random intercepts were significant for both automaticity, variance *u*_0__automaticity_ = 1.92*** [1.37, 2.69], and motivational interference, variance u_0automaticity_ = 1.09*** [0.78, 1.51]. Level 2 (L2) = habit-level; Level 1 (L1) = habit repetition level. 95% confidence intervals are in brackets. **p < 0.01, ***p < 0.001.

To test H2d, we conducted a multilevel mediation analysis using the MLmed Beta 2 macro by Rockwood (downloaded from https://njrockwood.com/mlmed; Rockwood, 2017). As in Models 6 and 9, we used the lagged HSE predictor variable HSE*_*t*_*_–__1_ and specified random intercepts. As expected, automaticity mediated the influence of HSE*_*t*_*_–__1_ on MI. We found this effect on Level 1 (within-indirect effect), *b* = −0.081 (*SE* = 0.011, *CI* = −0.10, −0.06), *p* < 0.001, as well as on Level 2 (between-indirect effect) with averaged Level 1 data, *b* = −0.123 (*SE* = 0.046, *CI* = −0.22, −0.03), *p* < 0.01. The CIs for the indirect effects were estimated using Monte Carlo simulations with 10,000 samples. See Figure 2 for the complete mediation analysis.

### Short Discussion

Study 2 generated three main findings. Firstly, we found evidence for a positive feedback loop in which HSE and automaticity amplify each other in the habit building process. Secondly, HSE predicted a reduced experience of MI during future habit performances. Thirdly, the effect of HSE on the experience of MI was partially mediated by automaticity. The analysis of this field data provides insight into the mechanics of habit development with a broader variety of freely chosen habits and participant characteristics (e.g., international user base, balanced gender ratio, and greater age span) than in Study 1. By tying SE to the habit (HSE) rather than the person (GSE) and tracking its changes over time, we found evidence for SE playing a central role in newly forming habits because of its interactions with automaticity and an attenuating effect on the experience of MI during habit performance.

## General Discussion

In this article, we explored the role of SE in the habit building process by applying hierarchical growth curve modeling to time series data. Our evidence suggests that SE is fundamentally beneficial for habit building (i.e., automatization of behavior). We could find connections between SE and automaticity in both analyzed datasets: Participants of Study 1 high in GSE ended up with stronger habits, while real-life app users’ lagged HSE predicted automaticity in future habit repetitions in Study 2. Furthermore, we found that lagged automaticity could in turn predict HSE. With both lagged HSE and automaticity predicting each other, we found evidence for a self-amplifying virtuous cycle of HSE and automaticity in habit building.

Concerning MI during habit performance, however, the specificity level of SE seems to matter. While GSE failed to predict MI in the Study 1, lagged HSE predicted reduced MI during habit performance in Study 2. Finally, we found direct and indirect effects of HSE on MI mediated by automaticity both on Level 1 (habit repetition) and Level 2 (aggregated habit-level values), meaning that HSE could predict MI on a granular level on specific habit repetitions (Level-1-effect) and HSE averaged over habit repetitions could predict average MI (Level-2-effect).

Since the breakthrough of smartphones, tracking habit development over time with an event sampling approach has become a lot more feasible. Next to high ecological validity when collecting data in real-life settings, time series data allows growth curve analyses that can provide new insights in the dynamic habit-forming process. The user dataset we analyzed has two additional advantages over the study dataset. Firstly, there was no extrinsic reward for logging habit data (the participants of the study dataset could receive course credit and participated in a lottery for an iPad) and, secondly, the habits were not constrained to study related behavior. The replication of the typical automaticity growth curve with steep automaticity gains in the earlier repetition range and asymptotically slowing gains in the higher repetition range with this broad range of behaviors shows how universal and robust the underlying principles of habit development ostensibly are. When people choose to build beneficial habits in order to achieve valued long-term goals, many of them seem to be able to do so with a little guidance during the habit definition process and simple feedback on their automatization progress.

### Be Specific When Using Self-Efficacy

General self-efficacy and HSE are relatives, but not twins. HSE can predict experience of MI and GSE cannot. But there is always a tradeoff. GSE may predict a far broader range of outcomes due to its generality (e.g., self-regulation, self-esteem, optimism, and psychological quality of life, Luszczynska et al., 2005a). However, if we want to make domain- or task-specific predictions, we gain a lot of predictive power by specifying SE. Richardson et al. (2012) report strong meta-analytical proof for this predictive boost by specificity. GPA and the more general academic SE are only weakly to moderately correlated (*ρ* = 0.28), while the task specific performance SE is strongly tied to GPA (*ρ* = 0.67). Richardson et al. distinguish academic SE and performance SE in a way that it related to the distinction we make in this article between GSE and HSE. Academic SE relates to expectancies about unfamiliar challenges for which performance must be anticipated in a more general manner. Performance SE, on the other hand, is related to expectancies about more similar challenges – like repeating a study behavior in the context of habit building.

### Believing and Executing: The Virtuous Cycle of Self-Efficacy and Automaticity

We do our first habit repetition and finish it successfully. The next time, our new habit will be triggered, we remember the mastery experience of how we successfully performed it the last time and engage in the behavioral sequence with a positive expectancy about our ability to finish it. So, we do it again and again, building automaticity, which makes it easier and easier to perform our habit increasing SE along the way. But it is not a given that this cycle will always kick in the way we wish it would. We want to make three remarks on this and then extend our scope to the important field of health-promoting behaviors.

Firstly, the development of automaticity must be salient to increase SE. In order to form a SE belief, the informational source needs to be available in the mind. However, one of the characteristics of automaticity is non-awareness (Bargh, 1994). There are two processes that have probably triggered reflection and thus salience of increased automaticity: The mere answering of the automaticity items after each habit repetition and the prominently displayed a graph of the automaticity growth curves of each habit to visualize habit growth. The pondering about how automatic one’s habit has already become and the feedback about it might have boosted – as intended – HSE development. Thus, when habits form in real life, the virtuous cycle of SE and automaticity might be weaker without these nudges to contemplate about developed automaticity.

Secondly, when we overburden ourselves with a very complex, big habit right from the beginning, it is more likely to book losses and decrease SE which will in all likelihood lead to abandon the habit eventually, even though substantial amounts of desired behavior might have been performed. When a student learns three pages of a textbook and then quits but set out to learn 10 pages with each habit repetition, she would frame this episode as a failure, thereby decreasing her SE for future repetitions. Simply lowering the habit repetition goal could enable the virtuous cycle to kick in. Once automaticity and SE are sufficiently developed, the habit repetition goal can be increased without the danger of overloading the newly formed behavioral frame.

Finally, traits holding back SE development could stifle habit building potential. GSE is linked to higher self-esteem, an internal locus of control, and less neuroticism (Judge et al., 2002). SE and high anxiety, depression, neuroticism are negatively correlated (Muris, 2002). These results are merely correlative and the direction of causality is unclear. However, there is evidence that self-esteem might influence SE formation, as low self-esteem individuals suffered higher SE loss after failure than high self-esteem individuals (Lane et al., 2002).

In terms of practical considerations, the virtuous cycle of SE and automaticity might also inform decision making related to health interventions. Many health-promoting behaviors such as a good diet and physical activity need long-term persistence to have positive effects (World Health Organization, 2020), but are often discontinued when motivation fades (Dombrowski et al., 2014). Even simple behavior like taking medication as prescribed is only done approximately 50% of the times (Brown and Bussell, 2011), causing detrimental outcomes like worsened diseases and death (Osterberg and Blaschke, 2005). When automaticity guides this health-promoting behavior (i.e., it has become a habit), it is more likely to be performed. Habit strength is associated with higher medical adherence rates (Badawy et al., 2020), physical activity, inactivity, as well as healthy and unhealthy diets (Gardner et al., 2011). In the *Behavior Change Wheel* model (Michie et al., 2011), a broad framework that allows classification of behavior change interventions, automatic behavior is a central source of behavior. As we showed in this article in the context of an app-supported, intentional habit building intervention, behavioral automatization seems to interact with more reflective HSE beliefs. When designing and evaluating interventions that rely on behavioral automatization, the virtuous cycle of automaticity and HSE should be considered. Habit forming interventions can have a broader influence beyond automaticity, which should be considered when measuring and evaluating the interventions. Furthermore, interventions designed to promote healthy behavior can deliberately focus on building HSE, as it facilitates self-regulation during the desired activity, but also supports automatization. Increasing HSE might be done in more ways than just with mastery experiences by successful behavioral repetition, such as verbal persuasion or vicarious experience (Bandura, 1994). However, more empirical data is necessary to test if these alternative sources of SE yield satisfactory increases in the special case of HSE.

### Limitations

Both samples consisted of young iPhone users and in Study 1, female psychology students are overrepresented. For both datasets the samples are comprised of people who are attracted to the idea of intentionally building a new, useful habit. Hence, the generalizability to contexts with different populations that might lack the intention to build a certain new habit, can be questioned. That could be the case, for example, when management tries to implement a new productivity enhancing habit by top-down directive or when a teacher instructs students to build study habits.

Furthermore, it has to be noted that comparability of the habits from the different datasets is somewhat reduced. In Study 1, there are only study habits with clear constraints, while users of the app could freely choose which habit to pursue, albeit with recommendations concerning duration and the other habit definition variables. That said, the fact that we found the same automatization patterns over time and the beneficial effect of automaticity on MI (the effect on MI for the study dataset is reported in Stojanovic et al., 2020) in both datasets over a variety of different habits, indicates generalizability and robustness of these effects.

### Further Research Questions

Successful habit building of desired habits increases HSE. Does this boost in specific SE increase GSE in some kind of higher order virtuous cycle? As we saw, GSE seems to be beneficial for automaticity in the habit building process. That way, building successfully one specific habit could lead to a higher order virtuous cycle in which future useful habits are created with more ease, nourished by HSE of past habits that might be long gone having left their trace in the person’s GSE.

Next to building habits, it is also valuable to explore the antecedents of discontinuing beneficial habits. For example, we found mixed results concerning habit pausing. In Study 1, there was no effect of not doing one’s habit for approximately 1–3 days from time to time. In Study 2, however, we found that pausing did slow down automatization. It has to be noted, that these analyses of habit pausing have a survivorship bias as only data from subjects who continued the habit building process after pausing can be included. The final “pauses”, which ended in discontinuing, could not be measured. Other important variables related to habit discontinuance are context stability (e.g., Wood et al., 2005) and how – or if – habit-related long-term goals are adapted once they are achieved and if strong beneficial habits might live on goallessly.

## Conclusion

While the self-regulatory benefits of good habits are unambiguously clear, longitudinal data on the process of forming habits is surprisingly scarce. With this research, we were able to share insights on how intentionally initiated, beneficial habits grow in a messy real-world setting. We hope that the evidence we found for the dynamic between automaticity and SE in the context of habit formation will support the development of effective habit-based interventions and trigger further habit research considering process and outcomes alike.

## Data Availability Statement

The raw data supporting the conclusions of this article will be made available by the authors, without undue reservation.

## Ethics Statement

The studies involving human participants were reviewed and approved by Bielefeld University Ethics Committee. Written informed consent for participation was not required for this study in accordance with the national legislation and the institutional requirements.

## Author Contributions

MS developed the idea and study design. MS created both versions of the iPhone application, conducted the data acquisition, performed the statistical analyses, and wrote the manuscript. AG and SF contributed to the final version of the manuscript and provided critical feedback, which helped shaping the theoretical foundation of the manuscript. SF supervised the project. All authors contributed to the article and approved the submitted version.

## Conflict of Interest

The authors declare that the research was conducted in the absence of any commercial or financial relationships that could be construed as a potential conflict of interest.

## Footnotes

1 We also conducted the analyses for this dataset excluding the total of *n* = 190 habit repetitions that were logged even after having completed 42 habit repetitions in order to be more transparent concerning data selection as suggested by Steegen et al. (2016). Results did not differ from those presented in the results section with the 190 habit repetitions included.

2 The demographic data about app use by country, gender, and age were taken from the google firebase console (the database backend of the app) and also contain the excluded cases with less than two habit repetitions and data from the first author as it was technically not possible to exclude specific cases from analysis.

3 The SRHI-items 2–11 were used. However, items 4, 6, and 11 were removed with one app update. Thus, 4 and 6 only had 39 measurement points each and item 11 had 110, while the rest of the items had contributed each between 727 and 834 measurement points. The average values of the automaticity scale with and without items 4, 6, and 11 correlated with *r* = 0.99, which is why we decided to keep the underrepresented items in the scale and not lose the data points.
